# Levodopa-Based Changes on Vocalic Speech Movements during Prosodic Prominence Marking

**DOI:** 10.3390/brainsci11050594

**Published:** 2021-05-04

**Authors:** Tabea Thies, Doris Mücke, Richard Dano, Michael T. Barbe

**Affiliations:** 1 Department of Neurology, Faculty of Medicine and University Hospital, University of Cologne, 50924 Cologne, Germany; michael.barbe@uk-koeln.de; 2 IfL Phonetics, Faculty of Arts and Humanities, University of Cologne, 50931 Cologne, Germany; doris.muecke@uni-koeln.de; 3 Institute for Nursing Science, Faculty of Medicine, University of Cologne, 50935 Cologne, Germany; richard.dano@uk-koeln.de

**Keywords:** speech production, prosody, speech kinematics, Parkinson’s disease, levodopa, articulation, vowel

## Abstract

The present study investigates speech changes in Parkinson’s disease on the acoustic and articulatory level with respect to prosodic prominence marking. To display movements of the underlying articulators, speech data from 16 patients with Parkinson’s disease were recorded using electromagnetic articulography. Speech tasks focused on strategies of prominence marking. Patients’ ability to encode prominence in the laryngeal and supra-laryngeal domain is tested in two conditions to examine the influence of motor performance on speech production further: without dopaminergic medication and with dopaminergic medication. The data reveal that patients with Parkinson’s disease are able to highlight important information in both conditions. They maintain prominence relations across- and within-accentuation by adjusting prosodic markers, such as vowel duration and pitch modulation, while the acoustic vowel space remains the same. For differentiating across-accentuation, not only intensity but also all temporal and spatial parameters related to the articulatory tongue body movements during the production of vowels are modulated to signal prominence. In response to the levodopa intake, gross motor performance improved significantly by 42%. The improvement in gross motor performance was accompanied by an improvement in speech motor performance in terms of louder speech and shorter, larger and faster tongue body movements. The tongue body is more agile under levodopa increase, a fact that is not necessarily detectable on the acoustic level but important for speech therapy.

## 1. Introduction

Parkinson’s disease (PD) is the second most common neurodegenerative disorder. Despite several non-motor symptoms, the main characteristics of the disease are related to gross motor but also speech motor problems. Impairments affect important aspects of daily activities, such as moving and speaking, which reduce patients’ quality of life. Therefore, therapeutic options seek to optimize motor functions and communication skills to increase a patient’s ability to participate in daily life [1,2].

Due to reduced control over muscles necessary for speech production, many patients develop a speech disorder, namely a hypokinetic dysarthria. This hypokinetic dysarthria affects all motoric subsystems of speech and leads to a reduced modulation of intensity and pitch, slower speech rate, imprecise articulation as well as an overall reduced articulation space [3,4]. Most studies on hypokinetic dysarthria are either based on perceptual intelligibility/ naturalness ratings [5,6,7,8] or acoustic speech analyses of standardized tasks, such as sustained vowel phonation, oral diadochokinesia (DDK) or a reading text [9,10,11,12,13].

So far, only a few studies examined underlying speech movements in the articulatory domain that determine acoustic speech properties (acoustic level) [14,15,16]. We can assume that temporal and spatial modifications of oral vocal tract actions underlie changes in syllable productions in Parkinsonian speech. Whereas temporal modifications relate to changes in movement duration (shorter or longer movements), spatial modifications refer to changes in the amplitude of a movement (smaller or lager displacements). Therefore, kinematic studies track movements of the jaw, lips, tongue tip and tongue dorsum during speech by using measurements such as electromagnetic articulography. Two studies investigated orofacial movements during natural sentence production in Parkinsonian speech [14,16]. They reported reduced jaw and lip movements in the temporal and spatial domain for patients when being compared to healthy controls. A third study, which is restricted to fast syllable repetition tasks (oral diadochokinesis of /papapa/), reported slower but not smaller lip movements [15]. So far, not much is known about tongue kinematics in Parkinsonian speech. There is one study that investigated tongue movements during the production of alveolar and velar consonants [17]. It compares dysarthric patients with PD, and non-dysarthric patients with PD and healthy controls. When comparing PD patients with and without dysarthria, temporal differences in terms of faster and shorter movements were found for the lingual consonants in the dysarthria group. However, when comparing patients with dysarthria to controls, patients performed with larger, longer and faster tongue movements. In contrast, patients without dysarthria produced smaller, slower, and longer tongue movements compared to healthy controls. This study questions the assumption of reduction in dysarthric speakers with PD in terms of consonantal weakening. Another study investigated lip and tongue movements during the consonant and vowel production of two patients with PD and two healthy controls, all of which are Italian native speakers [18]. The data show a very heterogeneous pattern on the articulatory level for the patient group ranging from hyper- to hypoarticulation within and across speakers. Interestingly, both PD patients showed consistent patterns for lip movements which are smaller in amplitude and shorter in duration when being compared with the healthy controls. In contrast, the tongue closing gesture was larger in the two patients than in the healthy controls.

Only one of the articulatory studies addresses the lingual movements during vowel production. This is surprising, since several acoustic studies already reported on reduced vowel spaces in Parkinsonian speech, and it is unclear how the reductions are related to speech motor control (and whether there are parallels to gross motor control). The present study aims to fill this gap and explores acoustic and articulatory parameters of vowel production by using electromagnetic articulography in patients with PD. In order to control for prosodic patterns that have been shown to influence the syllable internal coordination patterns, the study uses mini dialogues and avoids standardized tasks. Prosody is an important domain of speech production as it makes communication vivid, emotional, intelligible and more natural [5,19]. The ability to mark prominence (highlighting important words in a sentence) in the different domains of speech production (intensity, durational properties, pitch and vowel production) is tested in two conditions to examine the influence of motor performance on speech production further: without dopaminergic medication (bad motor performance) and with dopaminergic medication (improved motor performance due to levodopa intake).

### 1.1. Parkinson’s Disease

Bradykinesia, rigidity and a resting tremor are early motor signs manifested in patients with PD. In later stages, axial symptoms, such as postural and gait impairment, speech problems (hypokinetic dysarthria) and dysphagia develop. These motor problems are caused by deficits in the dopaminergic brain systems [1,20]. A decreased dopamine concentration within the basal ganglia affects brain activity in neural circuits responsible for voluntary movements. To improve motor performance again, patients with PD are treated with dopaminergic drugs, such as levodopa. Levodopa stimulates dopamine receptors in the brain and increases the dopamine concentration accordingly [1]. Movements of the limbs that were previously slow, small and stiff become larger, faster and less rigid. In addition, patients regain control over the initiation of movements. However, as tremor responds well to levodopa in only a few cases, this motor symptom is often treated with other pharmacologic agents. Moreover, axial symptoms seem to be less levodopa-responsive than non-axial ones.

PD develops very individually, but clinical subtypes can be defined in accordance with the most dominant motor symptoms. These are mainly divided into tremor-dominant and non-tremor-dominant types. The tremor-dominant type is characterized by a predominant resting tremor and a slower progression rate [1]. The non-tremor-dominant type can be further divided into an equivalent or akinetic-rigid subtype. Whereas an akinetic-rigid type would imply bradykinesia and rigidity, an equivalent type is affected in all three motor features. Moreover, some clinicians and scientists recommend that non-motor symptoms should also be taken into account when specifying subtypes of PD [21,22]. While a uniform categorization does not exist, patients are described in everyday clinical practice with regards to the types reported above.

### 1.2. Influence of Levodopa on Speech

Although it is proven that levodopa is an effective treatment for improving gross motor performance leading to faster and less rigid limb movements [23], it remains unclear to what extent it influences speech motor control. Only a few studies investigated the effects of levodopa intake on speech parameters validated with acoustic measures comparing medication-OFF status (patients stopped medication for at least 12 h) with a medication-ON condition. Most studies investigated glottal and articulatory functions in terms of fundamental frequency (*f*_0_) variation, intensity, speech rate (or articulation rate) and vowel articulation in terms of formant frequencies to capture the size of the vowel space. Interestingly, acoustic results across these different studies were often inconclusive. This indicates that we are dealing with a complex problem when investigating the relation of levodopa and speech performance. Some problems concerning comparability are likely due to the fact that the studies used very different speech tasks, in which prosodic structure is controlled in different ways (or even not controlled at all).

Studies recording sustained vowel phonation found that glottal control increases [24] but most often stayed unchanged [25,26,27,28,29,30] when comparing medication-OFF and medication-ON conditions. Moreover, many studies describe changes in speech rate with levodopa intake [26,27,28,29]. An exception is the study by De Letter et al. [30], which shows that the speech rate of DDK increased in patients with advanced PD with dopaminergic medication. In addition, a study by Ho et al. [31] reports overall increased intensity values, indicating changes in loudness due to levodopa intake.

Results on vowel production reveal quite different outcomes. In all studies, acoustic vowel analyses are based on the vowel formants F1 and F2. One study reported an increase in the overall vowel space area based on five corner vowels (/i, e, a, o, u/) in the medication-ON condition [32], reflected in a greater jaw opening for producing /a/, a more fronted and closed tongue position for /i/ and a retraction of the tongue for /o, u/. In contrast, another study found levodopa to have a deteriorating effect on vowel production, reducing the vowel space in the medication-ON condition [33]. Other studies measured the vowel articulation index based on the F1 and F2 of the vowels /i, a, u/ and reported no effect of levodopa on vowel formants [29,34].

The most relevant work from the perspective of the current study was carried out by Azevedo et al. [35], who investigated the influence of levodopa on the production of prosodic prominence. Ten patients with PD were asked to produce sentences in four different modalities: certainty, doubt, declarative and interrogative, which all require changes in prosodic parameters across the utterance. This work is an acoustic study, and the results indicate that patients are capable of modulating prosodic parameters such as acoustic duration, intensity and *f*_0_ with and without medication. The dopaminergic medication only induces shorter syllables on the acoustic level, while the other domains of speech production, i.e., intensity and *f*_0_, remained unchanged.

We already mentioned that kinematic studies on Parkinsonian speech are rare. However, there are two studies examining the influence of levodopa in the articulatory domain. A rather old study from the 1970s investigated lip movements by using an EMG study. Labial muscle activity was measured for seven patients producing sequences consisting of vowel—consonant—vowel [36]. Six out of seven patients performed with faster and smoother lip movements and improved articulation of stop consonants under levodopa treatment. A more recent study also investigated lip kinematics using a motion capture system recording DDK tasks performed by ten patients with PD [37]. In the medication-ON condition, syllable cycles of /papapa/ were performed with greater perioral stiffness, which further correlated with increased lip amplitude.

As we have shown, effects of levodopa on speech parameters remain rather inconclusive. It seems that previous research in the acoustic domain concludes that phonation and speech rate do not change, while there is evidence that intensity increases. It furthermore appears to be unclear whether the size of the vowel space changes or not with dopaminergic medication. In the articulatory domain, there is a clear lack of kinematic studies. So far, there is some evidence that—analogously to limb movements—the lips move faster and more flexibly with levodopa intake.

### 1.3. Prominence Marking

A lot of information is exchanged during a conversation. Depending on the conversational situation, some information is considered particularly important, while other information is considered less important. In these cases, prosody plays an essential role in conveying the meaning of an utterance [38]. On the one hand, prosodic prominence marking is a strategy to highlight important information. On the other hand, less important information is moved to the background, away from the listener’s attention. To highlight a word or phrase, the degree of its prominence is enhanced. Enhancing prominence in intonation languages such as German is achieved by changes in the laryngeal and supra-laryngeal system to accentuate specific syllables and realizing them more prominently than others [39].

Whereas changes in the laryngeal system relate to pitch movements, changes in the supra-laryngeal system refer to modifications of articulatory movements, as formulated in the Source-Filter Theory by Fant [40]. Laryngeal modifications involve changes in pitch values, especially on the stressed syllable of the target word [41]. Changes in pitch values produce different tones. These tones can be categorized into so-called pitch accents. Pitch accents affecting stressed syllables differ in their tonal movement, representing either a falling or a rising contour [42], but also on the tonal range. On the basis of perception experiments, pitch accent types with rising tonal movements are perceived as more prominent than falling movements [41,42], and the higher the tone, the higher the degree of prominence.

In addition to pitch changes, articulatory movements are adjusted in the spatial and temporal domain for achieving a more distinct articulation to increase contrasts in the acoustic and perceptual space further. To enhance prosodic contrasts, two strategies can be taken into account: sonority expansion and hyper-articulation [43,44,45,46,47,48,49]. Sonority expansion leads to a higher degree of opening of the oral cavity to allow for more radiation of acoustic energy from the mouth. This increase contrasts on the syntagmatic axis. Interestingly, longer acoustic vowel durations also lead to the impression of increased sonority. The localized hyper-articulation strategy is based on the H&H model [50] and involves more extreme vocal tract configurations to enhance paradigmatic contrasts such as place features in vowels. Therefore, the articulation is more distinct for producing prominence: In a prominent position, the front vowel /i/ is articulated with a more fronted tongue, and the low vowel /a/ with a lower tongue position, leading to more peripheral formant frequencies, which can be measured on the acoustic level. This means that, under prominence, the vowel space is systematically increased, which requires fine motoric adjustments of the oral articulators (especially the tongue dorsum).

Highlighting strategies for prosodic prominence are not restricted to *across-accentuation* involving the distinction between unaccented and accented syllables. Moreover, speakers encode prosodic prominence also *within-accentuation* in order to mark different degrees of contrast, e.g., broad focus vs. contrastive focus. Focus types are determined on the basis of the information structure of a word or phrase within a communicative context. While given information is classified as less important and thus not highlighted on the surface production, new or less accessible information is made prominent. Examples of the three focus types investigated in this study are the following:

(1) Background (*“a car” is given, not accented*):
  Q: *Does Max want to buy a car?—*A: ***Anne*** *wants to buy a car.*

(2) Broad focus (*whole utterance is of interest, accented*):
  Q: *What’s new?—*A: ***Anne*** *wants to buy**a car**.*

(3) Contrastive focus (*“a car” is new and corrective, accented*):
  Q: *Does Anne want to buy a house?—*A: *Anne wants to buy **a car**.*

In the examples 1–3 of the given question-answer scenarios, the constituent of interest is “a car”, marked by underscore. Accented, prominent words are highlighted in bold. Typically, words in the background position are not realized with prosodic adjustments. Broad focus refers to a unit larger than a word in which one or two constituents receive prominence, as the whole answer provides new information. Contrastive focus is restricted to prominence on a single word (occasionally narrowed to a single syllable). As contrastive focus is also known as “corrective focus”, it is used to correct specific content by contrasting the right element to the previously introduced constituent (house–car). Prosodic adjustments increase from background to broad focus and further to contrastive focus production, as contrastive focus has been found to involve the greatest prosodic adjustments [51,52]. Moreover, tonal movements differ between the broad focus and contrastive focus conditions. While contrastive focus is produced with a steep rising pitch movement, broad focus may be produced with a falling pitch movement [53].

Prominence marking can be investigated on the acoustic level but also on the underlying articulatory movements of the lips or the tongue [51,52]. Mücke and Grice [53] investigated lip opening movements across- and within-accentuation in native German speakers. Measurements of lip kinematics reveal an increase in lip movement duration (longer), an increase in amplitude of the lip movement (larger) and an increase in movement velocity (faster), comparing either the background and contrastive focus conditions, or comparing the broad and contrastive focus conditions. Another study on German speakers by Pagel et al. [54], which investigated tongue gestures, found that tongue positions and velocities systematically change with prominence across- and also within-accentuation. Vocalic gestures for /a/ and /o/ are adjusted in the vertical dimension, e.g., greater jaw opening for /a/. For producing the vowel /o/ in the prominent position, tongue movements were also adjusted in the horizontal domain, e.g., retraction of the tongue to achieve a vocalic target on the periphery. Moreover, vertical movements were performed faster across-accentuation as well as within-accentuation for the vowel /a/. To sum up, gradient changes were found on the articulatory level between focus types encoding different degrees of prominence. The changes involved systematic modifications in the temporal and spatial domain (hyper-articulation and sonority expansion).

For patients with PD, previous research on prominence marking is based on acoustic speech data. It postulates that prominence marking can vary from speakers with PD compared to healthy controls [55,56]. Moreover, a recent study by Thies and colleagues [57] showed that patients with PD can mark prosodic prominence by modulating *f*_0_, intensity and vowel articulation in prominent positions, but the prosodic modifications are less efficient than in healthy control speakers. While patients performed with a reduced vowel space in terms of hypoarticulated vowels, they hyperarticulated prosodic parameters such as intensity and tonal range. However, the results are restricted to patients with regular medication intake.

The aim of the present study is to investigate the prominence marking strategies of patients with PD in two conditions to examine the influence of motor performance on speech production further: with dopaminergic medication and without dopaminergic medication. Furthermore, to contribute to the knowledge of changes in speech movements, not only acoustic data but also articulatory data of tongue movements are analyzed. No previous research investigated underlying articulatory adjustments to acoustic measures with regard to prominence marking and levodopa intake.

The hypotheses underlying this study are divided into the influences of levodopa and the strategies of prominence marking:

Effect of levodopa:

(1) Gross motor performance increases with levodopa intake.
(2) Articulatory speech motor performance increases with levodopa intake, embodied in faster, larger and shorter (more flexible) movements of the articulators.
(3) Acoustic vowel space does not change with dopaminergic medication.
(4) Further acoustic speech parameters such as f0 modulation, intensity and vowel durations do not change.

Prominence marking:

(5) Patients with PD can manipulate f0, intensity and acoustic vowel durations in accented syllables to express local prominence [35,57].
(6) Patients with PD adjust the acoustic vowel space to signal prominence (hyper-articulation of vowels) [57].
(7) In prominent positions, articulatory tongue body movements (related to vowel production) are longer (increase in movement duration) and larger (increase in movement amplitude) [54].

## 2. Materials and Methods

### 2.1. Participants and Assessments

Sixteen patients with idiopathic Parkinson’s disease (11 male, 5 female) aged between 51 and 79 years participated in the study (Table 1). Inclusion criteria were a clinical diagnosis of the idiopathic Parkinson syndrome and German as the native language. Exclusion criteria were other preexisting neurological diseases such as a stroke and a history of previously induced speech problems. Assessment by a speech therapist excluded the presence of other speech and language problems such as aphasia, apraxia of speech or developmental disorders. None of the patients had clinically relevant signs of cognitive impairment, depression or dementia. This was assessed with the Mini-Mental State Examination (MMSE [58]), the Parkinson neuropsychometric dementia assessment (PANDA [59]) and the Beck Depression Inventory (BDI-II [60]). No restrictions were applied to disease duration or severity of dysarthria. However, all patients came to the clinic to learn about other therapeutic options beyond levodopa therapy, considering deep brain stimulation. This means that most of the patients were at an advanced stage of the disease and were already suffering from motor fluctuations. Furthermore, they already experienced inefficacy or lack of therapeutic success with levodopa therapy. Some patients did not suffer from fluctuations, but from severe, medication-resistant tremor.

The experiment took place in the department of Neurology at the University Hospital Cologne, Germany. Participants were tested during an in-hospital stay and gave a written informed consent before participating in the study. The study was approved by the local ethics committee of the University Hospital of Cologne (18–425).

Acoustic and articulatory recordings of speech data were carried out simultaneously with a 3-D Electromagnetic Articulograph (AG 501, Carstens Medizinelektronik GmbH, Bovenden, Germany). The acoustic signal was captured using a condensor microphone headset keeping the mouth-to-microphone distance of about 7 cm constant during the whole recording session. As gain levels were adjusted from medication-OFF to medication-ON recordings and between the participants, a reference tone for calibration of the intensity measures was recorded as the first stimulus in each recording condition. A detailed description of this procedure is available in Appendix A. The acoustic signal was recorded at 44.1 kHz/16 bit. To capture kinematic data, sensors were placed on the (1) lower lip, (2) upper lip, (3) tongue body and (4) tongue tip. The tongue sensors were placed approximately 1 cm and 4 cm from the beginning of tongue tip. Further sensors were placed behind the ears and on the nose for head correction. A test phase was included at the beginning of the experiment to allow the subjects to get used to the sensors. During this phase, all target words were produced in isolation by the participants (plus three practice trials).

Motor performance of the participants was evaluated using part III of the “Unified Parkinson’s Disease Scale” (UPDRS, [62]), a standard assessment for monitoring the motor ability of PD. Motor signs were rated on a 0–4 scale (0 = normal, 4 = severe). The maximum score that can be rated for this part is 108. A comparison of the motor scores in both conditions determines the influence levodopa had on the patients’ gross motor ability. The levodopa response indicates the percentage of motor improvement from the medication-OFF (med-OFF) to the medication-ON (med-ON) condition. To classify the PD subtype further, the tremor and non-tremor score was calculated and interpreted according to Eggers et al. [63].

Assessments were performed in a fixed order and started in the med-OFF condition, followed by the med-ON condition (Table 2). The reason why the order was not randomized is that the defined “OFF” state is achieved after abstaining 12 h from levodopa. Therefore, the patients received the last dose of medication at 6pm on the evening before the experiment. In order to receive the med-ON condition, patients received 200 mg of soluble levodopa (Madopar LT) before pausing. Then, 30–45 min after drug intake, the second test session started. In each condition, the visual recording for motor assessment was made first, followed by the speech recording.

### 2.2. Speech Material

**Figure 1:**
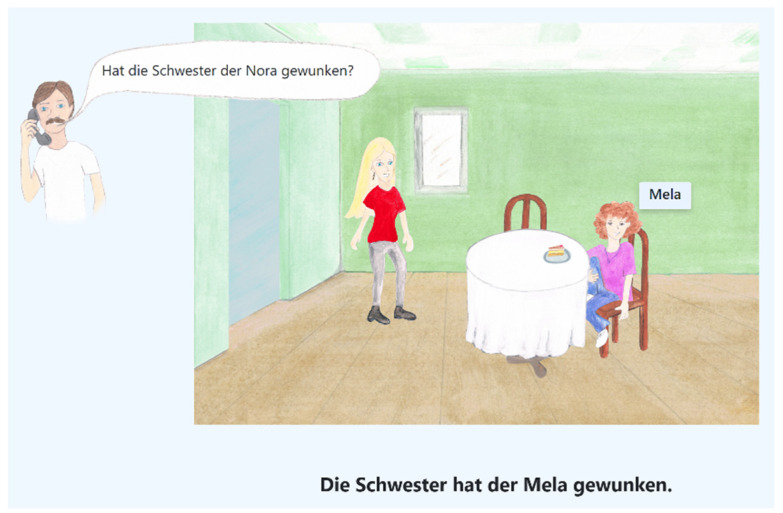
Game-like scenario to elicit target words.

**Figure 2:**
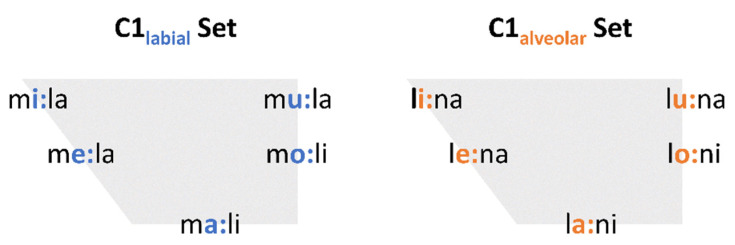
Target words divided into two sets depending on C1.

The speech task was designed as a question-answer scenario to elicit target words in a randomized order in three different focus structures: background, broad focus and contrastive focus (Figure 1). The questions were presented auditorily. The target words were 12 different disyllabic girls’ names (C_1_V_1_.C_2_V_2_) with word stress on the first syllable (Figure 2). Target words were embedded in a predefined sentence structure, such as: “Die Schwester hat der Mila gewunken” (*The sister waved to Mila)* or “Der Opa hat die Mali verlassen” (*The grandpa has left Mali)*:

(1) Background (*girl’s name is already given, not accented*):
  Q: Hat die Schwester der Mila gewunken? (*Has the sister waved to Mila?*)
  A: Die **Oma** hat der Mila gewunken. (*The grandmother waved to Mila.*)

(2) Broad focus (*whole answer is of interest, girl’s name is new, accented*):
  Q: Was ist passiert? (*What happened?)*
  A: Die **Oma** hat **der Mila** gewunken. (*The grandmother waved to Mila.*)

(3) Contrastive focus (*girl’s name is new and name corrected, accented*):
  Q: Hat der Opa die Mali verlassen? (*Has the grandfather left Mali?*)
  A: Der Opa hat **die Loni** verlassen. (*The grandfather left Loni.*)

To control for segmental context, some restrictions were made for the segments of the target words:

- C1 was either a labial or alveolar sonorant /m, l/
- V1 was one out of five peripheral vowels in German /i:, e:, a:, o:, u:/
- C2 was an alveolar sonorant: /l/ if C1 was labial; /n/ if C1 was alveolar
- V2 was either /i/ or /a/

To investigate the movements of the tongue body properly, vowel height was alternated not only in the stressed syllables but also in syllables immediately preceding and following the stressed syllable (e.g., CV_0_#CV_1_CV_2_ = /i-a-i/). In total, 960 tokens were included in the analysis: 10 words × 3 focus conditions × 2 medication conditions × 16 speakers. Each token was produced once per condition. No repetitions were made in order not to prolong the duration of the experiment with regard to the motor condition of the patient. Only utterances that were produced incorrectly were repeated.

### 2.3. Data Processing and Measurements

**Figure 3:**
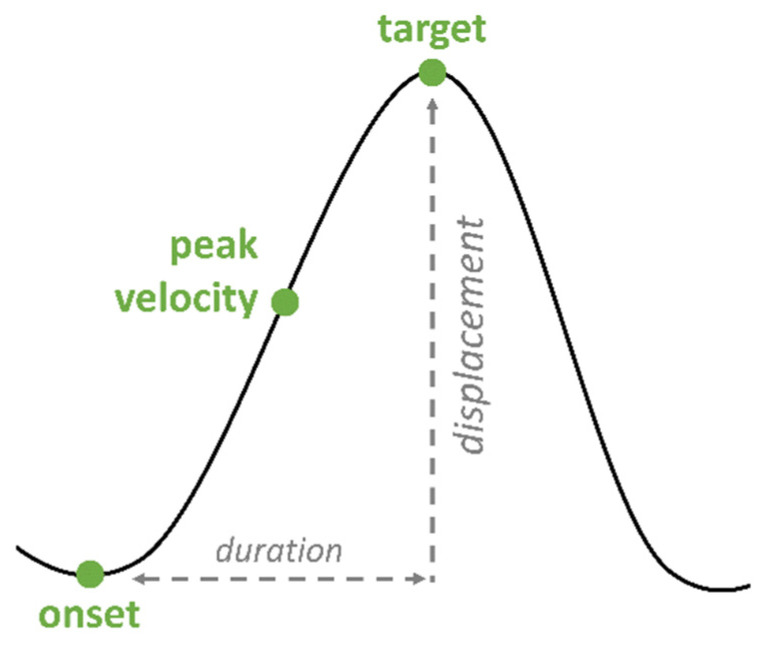
Articulatory landmarks of a schematized gesture.

The speech data were displayed and annotated using the EMU-webApp [64]. Target words, stressed syllables and the respective segments were determined according to the speech waveform and the wide-band spectrogram by inspection of the higher formant structure. As it is not always easy to identify segmental boundaries between sonorous consonant sounds, such as nasals (/m/) or laterals (/l/), and full vowels, a higher formant structure was used as the main label criteria. In laterals and nasals, the higher formants are considerably reduced in intensity [65]. In addition, nasals are identified by nasal formants. For the articulatory gestures of the tongue body, three landmarks were annotated in the vertical plane: onset, target and peak velocity (Figure 3). Onset, target and peak velocity were labeled by zero-crossings in the respective velocity and acceleration trace. The following prominence markers on the acoustic level were calculated:

*Acoustic vowel duration (ms):* Temporal interval between the start of the first vowel V1 and the end of the first vowel V1 of each target word. Longer vowels are associated with an increase in prominence.

*Tonal range (st):* The frequency difference between the starting point and the (high) target point of the *f*_0_ movement occurring in the vicinity of the first stressed syllable of the target word. Positive values indicate a rising *f*_0_ movement, negative values a falling *f*_0_ movement. An increase in tonal range is associated with a higher degree of prominence.

*Intensity (dB):* The mean intensity of the vocalic segment V1 was computed in relation to a reference tone to control for speaker variation. Increased intensity values are associated with an increase in prominence marking.

*Vowel Space and Vowel Articulation Index (VAI):* Based on the V1 vowel formants F1 and F2 of the vowels /i, a, u/, the Vowel Articulation Index was calculated using Equation (1), [66]. This measure reflects vowel contrast and vowel centralization and has been shown to be sensitive to highly variable data and dysarthric speech. Higher values represent an enhancement of the vowel space as expected during prominence marking (hyper-articulation).

*VAI* = (F2_i + F1_a)/(F1_i + F1_u + F2_u + F2_a) (1)

The articulatory analysis is limited to the production of the first vowel V1 as the main domain of focus production. Tongue body movements were investigated by including the following articulatory variables (Figure 3, [67]).

*Vocalic gesture duration (ms):* Temporal interval between the start of the vocalic gesture (onset) until the gestural target. An increase in gesture duration (longer gestures) is associated with an increase in prominence.

*Displacement (mm):* Spatial difference (displacement) between onset and target of the gestural movement. Higher displacements are associated with the production of more peripheral vowels, leading to an increase in prominence.

*Peak velocity (mm/s):* Maximum velocity of the movement. Faster movements are associated with an increase in prominence, even though the role of peak velocity is inconclusive in the literature [54].

**Figure 4:**
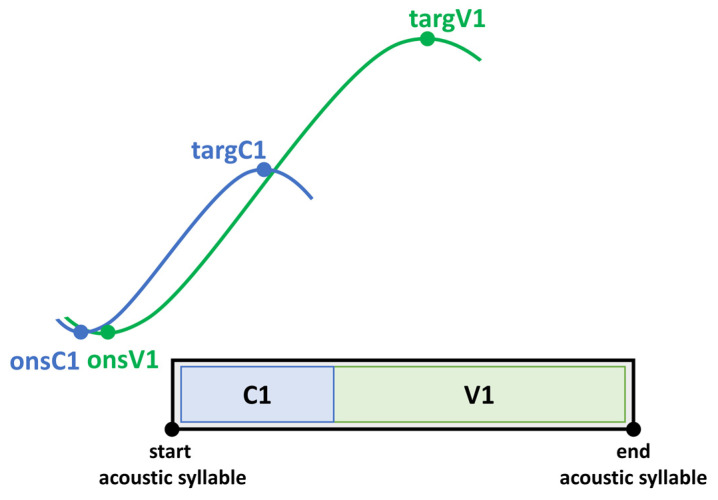
Coordination of articulatory gestures and acoustic segments within a CV syllable.

To capture the coordination between underlying tongue kinematics and speech properties on the acoustic level, coordination patterns between landmarks of the vocalic gesture and the acoustic syllable output are calculated. The expected coordination (temporal relationship) between articulatory gestures and acoustic segments is depicted in Figure 4. Note that articulatory gestures overlap in time (coarticulation). In a CV syllable, the consonantal and the vocalic movement start at the same time (onsC1, onsV1). Even though consonantal and vocalic movements in a CV syllable are activated at the same time, the vocalic gesture takes longer to reach its target (targV1). According to this, vowels have a longer movement duration. On the acoustic and perceptual level, one gets the impression of a CV sequence. Therefore, the start of a movement on the articulatory level does not correspond to the start of a segmental boundary on the acoustic level [68,69,70,71,72].

As the vowel is the most sonorous element in the syllable it is therefore the most important domain for prominence-induced changes within the stressed syllable. Coordination patterns were calculated for the gestural onset (onsV1) and the gestural target (targV1) of the vocalic tongue body movement with respect to the acoustic syllable. Negative values indicate that the landmark lies before the acoustic syllable. Positive values indicate an achievement within the acoustic syllable.

*Onset V1 to start of acoustic syllable (%):* Interval between the onset of the vocalic tongue body movement (onsV1) and the left acoustic syllable boundary (start) divided by acoustic syllable duration of the stressed CV syllable.

*Target V1 to start of acoustic syllable (%):* Interval between the target of vocalic tongue body movement (targV1) and the left acoustic syllable boundary (start) divided by acoustic syllable duration of the stressed CV syllable.

For calculating the percentage change of the UPDRS III motor score from the med-OFF to the med-ON condition, the following calculation was applied (Equation (2). In this equation, V_2_ represents the UPDRS III score in the med-ON condition, and V_1_ the one in the med-OFF condition. The same calculation was applied to measure the response for axial symptoms only (UPDRS III: Item 18, neck rigidity, Item 27, Item 28, Item 29, Item 30, [73]).

*Percentage change* = (V_2_−V_1_)/V_1_ × 100 (2)

### 2.4. Statistical Analysis

The main analysis is based on linear mixed effect models calculated with the lme4 package [74] in R Studio (RStudio Team, Version 1.4.1106). The analysis focuses on the relationship between z-transformed values of each continuous dependent variable and the critical predictors (medication condition, prominence condition) to investigate the effect of focus and levodopa. The parameters “vowel V1” and “consonant C1” were also included in the model as fixed factors. Random structure was added for “word per patient” to take into account the individual response of each patient to the target words. Models were validated by comparing the test model (with the critical predictor) to a reduced model (without the critical predictor) via likelihood-ratio tests. *p*-values are based on these comparisons. To correct for multiple testing, the Dunn–Šidák correction was applied, which lowers our analysis alpha level to *p* = 0.01274 for the acoustic data and *p* = 0.01695 for the articulatory data. Since the VAI calculation works across vowels and words, these two parameters were removed from the model structure for the statistical analysis.

One correlation between vocalic gesture duration and disease duration was calculated, for which it was previously tested whether these two variables were normally distributed. Since they were, the Pearson correlation coefficient is reported.

## 3. Results

### 3.1. Motor Assessment

The patient cohort primarily represents the akinetic-rigid and equivalent subtype of the Parkinson’s syndrome: seven of the patients are classified as akinetic-rigid, eight as equivalent, and one as tremor-dominant. The general motor performance was evaluated with the UPDRS III per condition. The averaged UPDRS III value in med-OFF was 34.69 (±10.8), ranging from 16 to 50. The value in the med-OFF condition differs significantly from the mean value in the med-ON condition (19.19 ± 6.4, ranging from 8 to 29), as a paired t-test indicates (t(15) = 6.9088, *p* < 0.001; d = 1.73). The motor performance improved on average by −41.72% (±20.5%). Referring to the arbitrarily set threshold of 30%, 3 of the 16 patients can be considered “non-responders” [75]. Axial symptoms improved on average by 27.97% from 6.2 (±2.7) in med-OFF to 3.8 in med-ON (±1.6), (t(15) = 3.507, *p* < 0.01, d = 0.88).

### 3.2. Speech Assessment

#### 3.2.1. Acoustic Results

**Figure 5:**
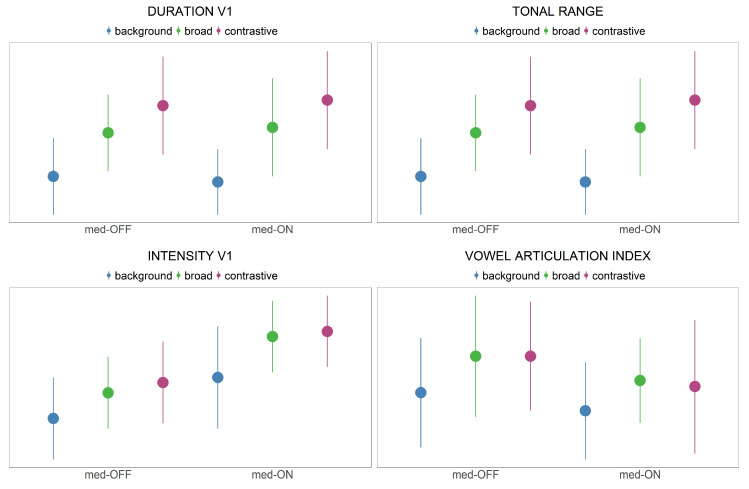
Presentation of z-transformed values (mean and sd) of acoustic measurements depending on medication condition and prominence condition.

Acoustic results are presented in Figure 5. Raw values are shown in Table 3, while Figure 5 depicts z-transformed values of the relationship between the medication condition and the focus conditions.

Acoustic vowel duration: The duration of the vocalic segment constantly increases from the background to broad to contrastive focus condition independent of medication condition (X^2^(2) = 152.27, *p* < 0.001). With levodopa intake, vowel durations do not change (X^2^(1) = 7e-04, *p* > 0.05).

Tonal range: The tonal range of the *f*_0_ movement constantly increases from the background to broad to contrastive focus condition (X^2^(2) = 486.15, *p* < 0.001). No difference was found between the medication conditions (X^2^(1) = 1.923, *p* > 0.05).

Vowel intensity: Intensity values increase comparing the background and broad focus conditions, but remain the same between broad and contrastive focus. The statistical model confirms the differentiation between non-prominent and prominent productions (X^2^(2) = 180.38, *p* < 0.001). Moreover, the model reveals an effect of medication (X^2^(1) = 296.55, *p* < 0.01) indicating that intensity values significantly increase from the med-OFF to med-ON condition.

**Figure 6:**
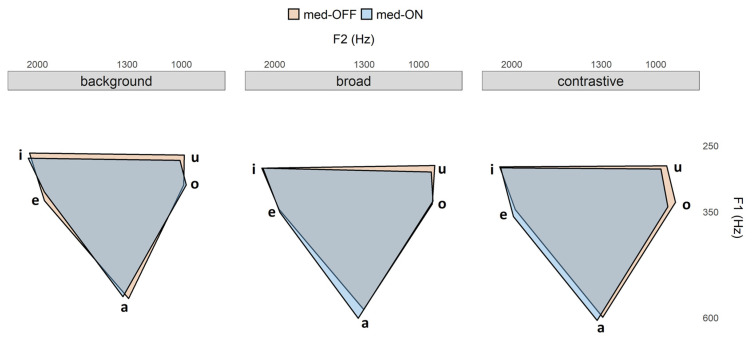
Vowel space area per prominence condition. Medication condition is indicated by color: med-OFF (orange) and med-ON (blue).

Vowel articulation index: The statistical model reveals that VAI neither changes according to focus condition (X^2^(2) = 6.1979, *p* > 0.05), nor to levodopa intake (X^2^(1) = 5.04, *p* > 0.01). To understand this issue better, the vowel space is mapped in Figure 6 as a function of the conditions.

To sum up, patients mark prosodic prominence in terms of acoustic parameters in the med-ON and med-OFF conditions. The analysis reveals a systematic increase of segmental vowel duration and tonal range across- and within-accentuation. Furthermore, there is a considerable increase in intensity from the unaccented to accented conditions (background to broad focus), while intensity remains the same between the broad and contrastive focus conditions. Interestingly, no spatial modifications of the vowel space were found at all, neither across-accentuation nor within-accentuation. The vowels are not hyperarticulated in the spatial domain to mark prosodic prominence. Only durational effects of prominence were found. Except for intensity, no group effects were found between the med-OFF and med-ON conditions in the acoustic exponents under investigation.

#### 3.2.2. Articulatory Results

**Figure 7:**
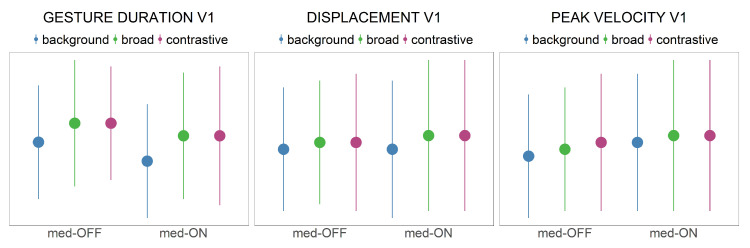
Presentation of z-transformed values (mean and sd) of articulatory measurements depending on medication condition and prominence condition.

Articulatory results are presented in Table 4 and Figure 7. Whereas raw values are shown in Table 4, Figure 7 depicts z-transformed values of the relationship between the medication condition and the focus conditions.

Vocalic gesture duration: The gesture duration increases from background to broad but does not continue to increase for contrastive focus production (X^2^(2) = 39.044, *p* < 0.001). Comparing the med-OFF to med-ON conditions, the statistical model reveals shorter gestures in the med-ON condition (X^2^(1) = 15. 684, *p* < 0.001).

Displacement: The gestures’ amplitude enhances from background to broad focus (X^2^(2) = 25.411, *p* < 0.001). No further increase from broad to contrastive focus is found. Comparing the med-OFF to med-ON conditions, the statistical model reveals higher gestural amplitudes in the med-ON condition (X^2^(1) = 7.576, *p* < 0.01).

Peak velocity: Tongue body movements are faster comparing background to broad and contrastive focus condition (X^2^(2) = 9.1686, *p* = 0.0102). The statistical model reveals no further change in speed between broad and contrastive focus. Comparing the med-OFF to med-ON conditions, vocalic gestures are faster in the med-ON condition (X^2^(1) = 25.417, *p* < 0.001).

Summarizing the articulatory results, prosodic prominence is marked by systematic modulations of the tongue body movements in all temporal and spatial measures under investigation. Vocalic tongue body movements are longer, larger and faster. However, parameters differ only between accented and unaccented target words, but not between broad and contrastive focus. Levodopa affects the tongue body trajectories in all measurements, resulting in shorter, larger, and faster movements as well as overall more flexible movement patterns.

#### 3.2.3. Coordination Patterns

Results for coordination patterns of the onset and target of the tongue body movement with respect to the acoustic syllable properties are presented in Table 5. The target of the tongue body gesture is achieved at 64% of the syllable duration, independent of focus structure (X^2^(2) = 2.5028, *p* > 0.05) and medication condition (X^2^(1) = 0.1411, *p* > 0.05). In contrast, vocalic onset coordination differs according to focus structure (X^2^(2) = 9.3841, *p* < 0.01) and levodopa intake (X^2^(1) = 15.911, *p* < 0.001). The onset of the vocalic gesture shifts to the right, closer to the acoustic start of the stressed syllable for producing prominence. This shift is even stronger in the med-ON condition, resulting in faster movements and reducing the distance between the articulatory onset and the acoustic syllable start.

### 3.3. Tongue Body Flexibility

In this section, we will address the relation of acoustics and articulation in terms of vowel production. We showed that the patients are able to mark prosodic prominence on the acoustic level by systematically lengthening the vocalic segment with respect to all focus conditions (across- and within-accentuation), while the vowel space in terms of formant frequencies remained the same. No effect of levodopa was found for the acoustic vowel measures. In contrast, levodopa intake affects the kinematic trajectories on all temporal and spatial measures in terms of faster, larger and shorter tongue body movements. Under levodopa intake, the tongue body appears to be more agile and move more flexibly. The aim of this section is to combine acoustic results with articulatory results and to explain why levodopa effects were found on the articulatory but not the acoustic level. We will explain why acoustic durations can remain the same, while durational properties on the articulatory level change.

As described above, consonantal and vocalic movements overlap in time. On the perceptual level, we get the impression that we are dealing with a segmental string, but due to coarticulation, there are no clear segmental boundaries. A vocalic movement of the tongue body is initiated before the vowel segment. Therefore, articulation of the vocalic sound starts before it is manifested in acoustic properties with its typical formant patters and thus before it is perceived on the surface. Moreover, in CV syllables, vocalic movements are initiated shortly after the consonantal movement and therefore begin before the acoustic syllable starts [70]. The target of the vocalic movement is usually reached before the vowel segment shows the transition to the following consonant (Figure 4).

**Figure 8:**
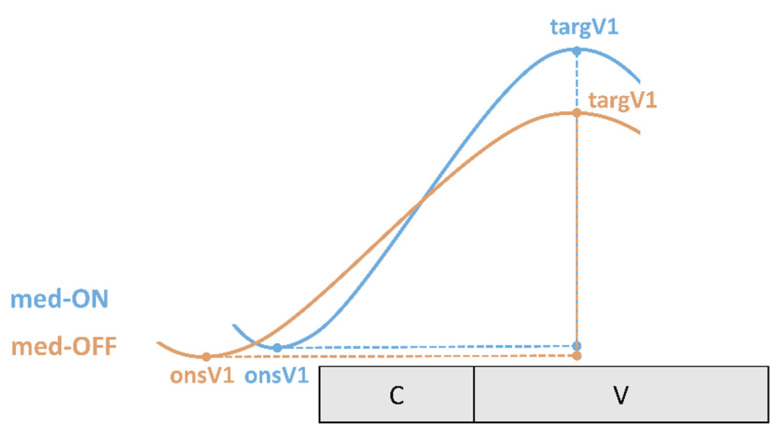
Schematized summary of results with respect to vowel articulation.

The schematized coordination patterns in Figure 8 demonstrate that acoustic durations remain the same, while tongue body movements are faster, larger and shorter in the med-ON condition compared to the med-OFF condition. That means that vocalic targets in the former are reached faster than in the med-OFF condition. However, this faster gesture does not reach the target earlier with respect to the syllable duration, as the vocalic target is consistently reached at 64% of the acoustic syllable. This further indicates that on the acoustic level, no changes appear. To explain why acoustic durations remain the same, while gestures become shorter in the med-ON condition, vocalic onset coordination must be considered. The underlying gesture can be shortened, because the movement is initiated later and therefore closer to the left boundary of the syllable on the acoustic level. Under prominence, there is a tighter internal syllable coordination, which is even strengthened under levodopa intake. Furthermore, it seems that patients with PD rely more on temporal modulation of parameters than on spatial adjustments.

**Figure 9:**
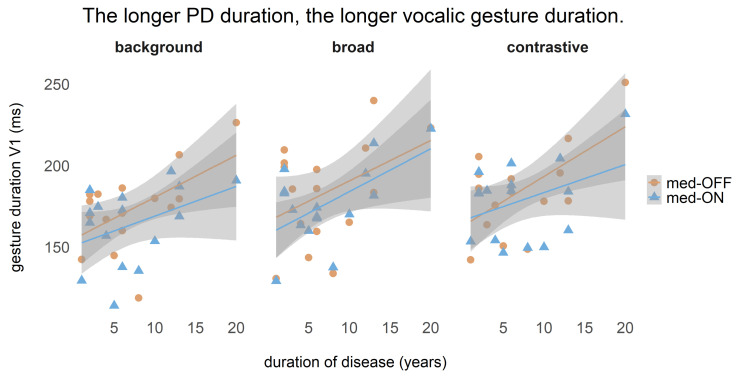
Correlation between vocalic gesture duration and disease duration per focus structure in med-OFF and med-ON conditions.

In the med-ON condition, patients with PD can control their tongue more flexibly. Nevertheless, compensatory mechanisms can be involved, which help to keep the acoustic target stable in the med-OFF and med-ON conditions. As the data show, patients with PD are able to produce the same acoustic output by varying the articulatory strategy to achieve the acoustic target. We assume that patients learn to cope with speech motor deficits as the disease progresses. To verify this assumption, a correlation between the vocalic gesture duration and the disease duration of patients with PD was computed. The results for each focus condition but also independent of the medication condition reveal that there is a strong relationship between disease duration and gesture duration (Figure 9). The longer the disease duration, the longer the duration of the tongue body movement. In the med-OFF condition, the correlation coefficients are the following: background: r = 0.54, broad: r = 0.41, contrastive: r = 0.60. In the med-ON condition, the correlation coefficients are on average lower: background: r = 0.40, broad: r = 0.57, contrastive: r = 0.38. This shows again that vocalic gesture durations are shorter in med-ON, and speech motor state improves with levodopa intake. However, it also indicates that patients with PD learn to cope with and compensate for motor problems on a linguistic level by increasing the duration of gestures in the course of the disease.

## 4. Discussion

The aim of this study was to investigate strategies of prosodic prominence marking in patients with PD on the acoustic and articulatory level. As speech production may depend on the overall motor ability, the influence of levodopa, a drug which improves the motor ability in patients with PD, was tested. To capture possible speech changes due to levodopa treatment, patients were recorded in two conditions: without an effect of medication and with intake of the drug “levodopa”.

Prominence marking: The collected speech material was used to investigate the modulation of prosodic parameters across three different focus conditions. In line with previous studies [34,55], the data indicate that patients with PD are able to encode prominence on the acoustic and articulatory level. As assumed in the fifth hypothesis for acoustic parameters, patients with PD modulate *f*_0_, vowel durations and intensity. They produce systematic contrasts between within- and across-accentuation in terms of longer vowel durations and higher *f*_0_ rises (background < broad < contrastive). Intensity is only adjusted across-accentuation to differentiate between accented and unaccented target words (background < broad = contrastive). This is in line with highlighting strategies reported for prosodic prominence in German [38]. As shown before by Thies et al. [57], hyper-articulation of vowels would be expected. However, no spatial modifications were found with respect to more peripheral vowel formant frequencies (no hyper-articulation). It is reported in the literature that patients exhibit a smaller vowel space than healthy controls. Having this in mind, the present study reveals that within this overall smaller vowel space, it is problematic to enhance the vowel space under prominence (Figure 6). This phenomenon is reflected in the VAI measure, which is based on vowel formants. The data indicate that vowels are not further contrasted when comparing prominent and non-prominent productions, so that no modulation/hyper-articulation is possible due to the reduction and centralization of the vowel space. When looking at the articulatory data, strategies for prominence marking are also found in the tongue body movements. In prominent positions, the vocalic movements produced were longer, faster and larger. This is in line with highlighting strategies reported especially for syntagmatic contrasts in terms of sonority expansion, since longer durations give the impression of an increase in sonority. Interestingly, the articulatory adjustments were found only between accented and unaccented target syllables, but not further between broad and contrastive focus.

It is important to note that there is no one-to-one relation between acoustics and articulation. Even though there were displacement differences in the kinematic trajectories to mark prosodic prominence, they were not reflected on the acoustic level (cf. [18]). While descriptively a spatial change (based on larger underlying tongue body movements) with respect to the different focus structure conditions can be observed in Figure 5 (bottom, right), the statistic does not reveal significant results for the measured vowel space values. It is possible to assume that the effects are too small to capture for this sample size in the acoustic domain. Actually, larger tongue displacements and increasing vowel spaces should go hand in hand, as shown by Mefferd [76]. However, the vocal tract is a complex filter system, and not all articulatory modifications induce the same spectral changes on the acoustic level. These nonlinearities between articulatory vocal tract configurations and acoustic output are captured by the Quantal Theory [77]. The QT predicts that there are regions in the vocal tract “(…) where perturbations in that parameter result in relatively small acoustic changes (e.g., in formant frequencies) and other regions where comparable articulatory perturbations cause substantial acoustic changes.” [78] (p. 72).

In addition to the study by Thies et al. [57], it is now becoming clear that the strategy of prominence marking does not change according to levodopa intake, as prosodic adjustments did not change between the med-OFF and med-ON conditions. Moreover, this study investigated a second focus condition, namely broad focus. By including this focus category in the analysis, it is possible to illustrate that the modulation of parameters differs. While clear differences between background and contrastive focus (the most prominent category) have previously been shown, the present study shows that the two categories of broad and contrastive focus merge into one on the levels of vowel articulation and intensity modulation. This pattern might be another strategy to compensate for the spatial speech deficits present in PD. Since control of vowel articulation and intensity modulation is limited, acoustic durations and *f*_0_ are modeled more strongly to establish prosodic contrasts. In particular, adjustments of durational properties and pitch increase the perception of prominence. Interestingly, we found a correlation between gestural durations and disease duration in that with increasing disease durations, prolonged vowels were produced. This indicates that the articulation rate decreased throughout the course of the disease—either due to increased motor severity (and problems to decouple the tongue dorsum from the jaw [79]) or as a strategy to deal with this speech deficit and to elaborate a way to achieve the same acoustic output, by achieving the articulatory target in a modified way.

Effect of levodopa: The overall motor performance improved in all patients by about 42% when comparing values of the UPDRS III in the med-OFF and med-ON conditions. Only three patients performed with a levodopa response below 30 and are therefore classified as non-responders [75]. These non-responding patients were diagnosed with PD 2–3 years prior to the study assessment. Two of them were later on implanted with a deep brain stimulation (DBS), with an indication of a tremor-dominant symptomatic (STN-DBS; VIM-DBS). The third patient did not received DBS. As expected, the levodopa response of axial symptoms was considerably lower, with 28%.

Focusing on the speech parameters, no effect of levodopa intake was found on the acoustic level for vowel production. However, a systematic group effect was detected between patients in the med-ON and med-OFF conditions in the respective articulatory domain. Under dopaminergic medication, the tongue body produced faster, larger and shorter movements. The tongue behaves more flexibly, and vocalic targets were reached in a more efficient way than in the med-OFF condition. This influences the syllable internal coordination pattern since vowels and consonants are not produced in isolation. As presented in Figure 8, articulatory coordination patterns differ between the med-OFF and med-ON conditions. Because vocalic gestures are articulated faster and in less time, they can be initiating later in the med-ON condition.

Whereas spatial modifications on the articulatory level were observable in the med-ON condition, the acoustic vowel space does not change with dopaminergic medication, which is in line with Skodda et al. and Jacobi et al. [29,34]. It is well known that the vowel space of patients with PD is reduced and centralized compared to healthy control speakers [11,57]. As for the effect of focus structure, acoustic changes in amplitude (within this reduced vowel space) were probably not strong enough to induce clear changes in the formant characteristics comparing medication conditions (trends for changes in vowel formants can be drawn from Figure 6). However, the more agile tongue movements underlying the acoustic speech output in the med-ON condition are relevant, as this improvement is reflected in the overall motor performance measured in the present study.

Furthermore, *f*_0_ modulations were not affected by levodopa intake, which is in line with the previous literature [24,25,26,29]. Although the control over the glottal speech system does not improve, increased intensity values were measured in the med-ON condition, indicating an improved subglottal pressure, as already investigated by Ho and colleagues [31]. This effect can be explained by an improved pulmonary function as well as stronger respiratory muscles, resulting in a better breath support and therefore in a global increase in intensity, leading to an overall louder speech output [80].

Limitations of the study: One explanation for the lacking levodopa effect on strategies of prominence marking might be based on task-dependent behavior [81]. The experimental set-up might be an efficient cue so that the patients are focused on the task and can retrieve speech motor performance to fulfill the requirements needed for prominence marking even when they are in the med-OFF condition. The same behavior can be observed for walking, which is also an axial symptom. In particular, auditory cueing seems to be effective for improving gait performance [82]. In our set-up, this would be equivalent to the questions which were presented auditorily in the question-answer scenario. In clinical settings, one can observe that when patients are asked to walk, or to perform a task, they can do this by using a reserve. Relatives also report, “if she/he wants, he/she can walk”. A study by Distler et al. [83] underlines that patients with PD can increase their movement velocity by reducing their bradykinesia under specific conditions. Critical factors for this phenomenon (paradoxical kinesia) are external sensory cues [84]. It seems that under certain circumstances, patients may deliver a performance that is generated selectively but cannot be maintained at a high level over the long term. Having this in mind, the results portray only a short-term effect, which may deteriorate over time.

Future Research: This study investigated short-term levodopa effects on speech production. A possible direction for future research could be to investigate the long-term levodopa effect to capture how general speech patterns but also compensation strategies change throughout the day with regular medication but also within a time interval of 6–12 months as the diseases progresses. Another option would be to investigate prominence marking strategies and levodopa effects comparing clinical subtypes. This would explain whether one subtype benefits more from levodopa intake and performs with increased speech motor control. Moreover, as this study did not include data of healthy control speakers, a direct comparison is missing. It would be interesting to compare articulatory speech data of a control group with patients with PD as well as a cohort prior to PD diagnosis to understand how and when underlying speech movements change and the vowel space reduces. Considering clinical implications of this study, it is now clear that patients are able to produce prominence but still show deficits within the vocal tract, especially tongue movements. Additionally, Mefferd and Dietrich found that patients with PD have a tongue-dominant articulatory impairment [79]. As articulation has the greatest impact on speech intelligibility, articulation should be the domain of speech therapy. Moreover, as a reorganization of the speech system is needed for compensation strategies, speech therapy could train on strengthening tongue muscles for increasing the vowel space in the end and further train the precision of articulatory movements. As suggested by Mefferd and Dietrich, slow speech could be a therapy option to train acoustic contrast and to maintain intelligibility. Depending on the motor state, therapists could focus on temporal modification to compensate for spatial deficits in a bad motor state, while for good motor state, spatial training might be further successful.

The patients increase the tonal range of *f*_0_ movements, intensity and vowel durations, while spatial proportions of the vowel space remain unchanged. For all acoustic parameters under investigation, only intensity was affected by levodopa. Interestingly, another picture arises when investigating the underlying movement patterns of the oral vocal tract. Tongue body movements are more flexible and agile with dopaminergic medication, resulting in shorter, faster and larger movements during the production of vowels. This is similar to gross motor skills, which also improved with levodopa intake. Patients with PD learn to compensate on the articulatory level to maintain the acoustic output. They predominantly use modifications in the temporal domain such as the lengthening of the vocalic movements to mark prominence in the phonetic substance. To sum up, changes in gross motor skills can also be projected onto speech motor performance and can be detected by using electromagnetic articulography.

## Figures and Tables

**Table 1:** Patients’ demographic and clinical characteristics. In addition, information about the PD subtype (AR = akinetic-rigid, E = equivalent, TD = tremor-dominant) and levodopa daily dose (LEDD [61]).

Patient	Gender	Age	Disease Duration (Years)	UPDRS III Med-OFF	UPDRS III Med-ON	Subtype	LEDD
						(Med-OFF)	
PD01	m	53	4	45	24	E	540
PD02	m	54	6	44	27	AR	720
PD03	m	58	2	20	11	TD	826
PD04	f	51	6	41	20	E	934
PD05	f	56	6	45	29	E	450
PD06	f	70	20	27	14	E	1400
PD07	m	63	10	35	16	AR	1197
PD08	m	65	3	20	20	E	300
PD09	m	60	8	48	19	AR	2311
PD10	m	56	1	30	16	E	300
PD11	f	56	2	16	15	E	420
PD12	m	68	12	36	8	AR	1158
PD13	m	54	5	26	12	AR	1030
PD14	f	69	13	41	27	AR	1387
PD15	m	79	2	31	27	E	700
PD16	m	69	13	50	22	AR	983
mean	-	61.3	7.1	34.69	19.19	-	916
(±sd)		(8)	(5.3)	(10.8)	(6.4)		(517)

**Table 2:** Procedure of the experiment.

UPDRS Video	med-OFF
Speech recordings	
200 mg levodopa intake and break	
UPDRS video	med-ON
Speech recordings	

**Table 3:** Means and standard deviations for acoustic parameters of interest depending on prominence condition and medication condition.

Parameter	Condition	Background	Broad	Contrastive
Vowel duration (ms)	med-OFF	120 (30)	130 (33)	134 (31)
	med-ON	116 (30)	130 (29)	136 (29)
Tonal range (st)	med-OFF	0.58 (1.5)	2.12 (1.9)	3.24 (2.2)
	med-ON	0.28 (0.9)	2.26 (2.4)	3.53 (2.6)
Intensity (dB)	med-OFF	72 (5)	74 (5)	74 (5)
	med-ON	76 (6)	78 (6)	78 (6)
VAI	med-OFF	0.94 (0.13)	0.98 (0.13)	0.99(0.12)
	med-ON	0.92 (0.13)	0.96 (0.08)	0.96 (0.08)

**Table 4:** Means and standard deviations for articulatory parameters of interest depending on prominence condition and medication condition.

Parameter	Condition	Background	Broad	Contrastive
Vocalic gesture duration (ms)	med-OFF	173 (40)	183 (46)	184 (41)
	med-ON	164 (40)	177 (45)	178 (44)
Displacement (mm)	med-OFF	7.4 (4.4)	7.8 (4.6)	7.9 (4.7)
	med-ON	7.6 (5.1)	8.4 (5.9)	8.3 (5.4)
Peak velocity (mm/s)	med-OFF	74 (43)	77 (42)	79 (44)
	med-ON	81 (50)	84 (51)	85 (51)

**Table 5:** Acoustic–articulatory relations: Coordination patterns (mean and sd) between the vocalic tongue body movement and the acoustic CV syllable. Negative values indicate that the landmark lies before the syllable. Positive values indicate an achievement within the acoustic syllable.

Coordination Pattern	Condition	Background	Broad	Contrastive
*Onset V1 to start of acoustic syllable (%)*	med-OFF	−22 (18)	−21 (16)	−20 (16)
	med-ON	−20 (19)	−18 (20)	−16 (16)
*Target V1 to start of acoustic syllable (%)*	med-OFF	64 (11)	64 (11)	63 (10)
	med-ON	62 (12)	64 (12)	64 (12)

## Appendix A

The procedure for calibrating the intensity values by using a reference tone is explained here. As patients with PD often speak louder in the med-ON condition, gain levels were adjusted from the med-OFF to med-ON recordings. Moreover, each patient had his/her own gain setting as some speak louder than others. A reference tone for calibration of the intensity measures was recorded as the first stimulus in each recording condition. The distance between the device playing the reference tone and the condensor headset was always the same. First, a mean intensity value was calculated for all references tones recorded in med-OFF. This is used as the reference value. Both the med-OFF and med-ON values of each speaker were then related to the calculated reference value. By doing so, a fixed factor for the med-OFF and med-ON conditions was measured, indicating either an increase or decrease in intensity for this condition. The degree of this change (as a factor) was then applied to all vocalic intervals of the session to get the relative intensity at the end. All intensity values were dragged to the same level per session and speaker. Therefore, all values were multiplied by the factorial change with respect to the reference value. As a last step, the intensity of the vocalic segment was determined.
